# Linking discrete and continuous models of cell birth and migration

**DOI:** 10.1098/rsos.232002

**Published:** 2024-07-17

**Authors:** W. Duncan Martinson, Alexandria Volkening, Markus Schmidtchen, Chandrasekhar Venkataraman, José A. Carrillo

**Affiliations:** ^1^ Mathematical Institute, University of Oxford, Oxford, UK; ^2^ Department of Mathematics, Purdue University, West Lafayette, IN, USA; ^3^ Institute of Scientific Computing, Technische Universität Dresden, Dresden, Germany; ^4^ Department of Mathematics, University of Sussex, Brighton, UK

**Keywords:** non-local interactions, zebrafish, self-organization, aggregation equations

## Abstract

Self-organization of individuals within large collectives occurs throughout biology. Mathematical models can help elucidate the individual-level mechanisms behind these dynamics, but analytical tractability often comes at the cost of biological intuition. Discrete models provide straightforward interpretations by tracking each individual yet can be computationally expensive. Alternatively, continuous models supply a large-scale perspective by representing the ‘effective’ dynamics of infinite agents, but their results are often difficult to translate into experimentally relevant insights. We address this challenge by quantitatively linking spatio-temporal dynamics of continuous models and individual-based data in settings with biologically realistic, time-varying cell numbers. Specifically, we introduce and fit scaling parameters in continuous models to account for discrepancies that can arise from low cell numbers and localized interactions. We illustrate our approach on an example motivated by zebrafish-skin pattern formation, in which we create a continuous framework describing the movement and proliferation of a single cell population by upscaling rules from a discrete model. Our resulting continuous models accurately depict ensemble average agent-based solutions when migration or proliferation act alone. Interestingly, the same parameters are not optimal when both processes act simultaneously, highlighting a rich difference in how combining migration and proliferation affects discrete and continuous dynamics.

## 1. Introduction

Self-organization of individual agents is a key feature of life. It occurs ubiquitously throughout the natural world, from the macroscopic example of bird flocking [1–4] to the microscopic phenomenon of cell sorting during development [5–9]. The degree to which members of a group coordinate their movement, proliferation and competition accounts for pattern diversity across biological scales. Alongside experimental approaches, mathematical models can help identify the underlying behaviours that give rise to specific collective dynamics. However, a trade-off often exists between tractability and detail when building models of pattern formation, due in part to the multiscale nature of biological systems. Consequently, better quantitative characterization of the relationship between analytically tractable models and more biologically representative approaches will improve our understanding of self-organization throughout nature.

**Figure 1.**
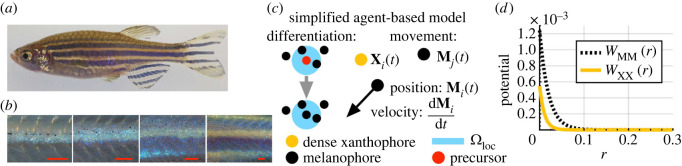
Motivating biological example and model. (*a*) Wild-type zebrafish feature stripe patterns in their skin. These patterns consist of several types of brightly coloured pigment cells. (*b*) Over the course of a few months, these cells organize sequentially into stripes and interstripes from the centre of the fish body outward [10]. (*c*) For the purposes of this paper, we focus on a single population of black melanophores or gold dense xanthophores, using a simplified version of the model from [14]. The agent-based model (ABM) [14] that motivates our work describes how patterns arise through cell differentiation, competition and movement. In our simplified version of the ABM [14], we assume new cells appear at randomly selected locations based on short-range activation; this models cell differentiation from uniformly distributed precursors (red position) [14], and we also refer to this as ‘proliferation’ or ‘birth’ in this paper. (We describe the cell differentiation rules in the full model [14] in more detail in electronic supplementary material, figure S1.) In both our work and the ABM from [14], cell movement is deterministic and governed by ordinary differential equations (ODEs). (*d*) These ODEs account for cell–cell repulsion through potential functions, which describe melanophore–melanophore ($W_{\mathrm{MM}}$) and xanthophore–xanthophore ($W_{\mathrm{XX}}$) interactions as a function of their pairwise distance *r*. Red scale bar is 250 μm in (*b*). Image (*a*) adapted from Fadeev *et al.* [15] and licensed under CC-BY 4.0 (https://creativecommons.org/licenses/by/4.0/). Image (*b*) adapted from Frohnhöfer *et al.* [10] and licensed under CC-BY 3.0 (https://creativecommons.org/licenses/by/3.0/); published by The Company of Biologists Ltd.

Here, we help address this open challenge using pigment cell dynamics in zebrafish patterns as a motivation. The zebrafish (*Danio rerio*) is a popular model organism for studying pattern formation, as dark stripes and gold interstripes emerge in its skin during development [10–13]. As we show in figure 1, these stripes result from the coordination of interactions among several types of cells, including black melanophores and gold (dense) xanthophores [10,16–20]. Experiments that perturb stripes—e.g. by laser ablation [16,21]—demonstrate how cell–cell signalling and external cues contribute to the creation of alternative motifs such as spots or labyrinths. A rich diversity of mutant patterns, including widened or curvy stripes, also emerge when cell interactions are altered due to genetic mutations [19,22].

Data-driven mathematical models can help uncover the drivers of zebrafish pattern formation and other biological phenomena exhibiting self-organization by identifying important phase transitions, isolating the effects of specific processes such as cell division, and providing hypotheses that can guide the design of *in vivo* experiments [23–26]. Different modelling frameworks yield insight at the population or individual level, depending on how they represent members of a group. One modelling approach involves tracking how the position of each individual changes in time. These so-called ‘discrete’ systems include centre-based models [27,28], cellular automata [29,30], cellular Potts models [31,32] and vertex models [33,34]. Within the setting of zebrafish patterning, agent-based models (ABMs) have been developed that restrict cells to occupy certain locations ‘on-lattice’ [35–37] or allow them to roam freely, ‘off-lattice’, in the domain [14,38–40]. Due to their ability to work on the same length scales as empirical data, ABMs provide an intuitive connection to experiments and allow for detailed predictions about how interactions between agents drive group behaviours. However, ABMs can be prohibitive to simulate when the number of individuals is large, and understanding their long-time behaviour under alternative rules and parameters relies on extensive computation [41].

A second modelling approach uses continuous functions to represent the ‘average’ density of agents in a collective, with their dynamics governed by a partial differential equation (PDE) in space and time. Continuous models, including reaction–diffusion equations, Boltzmann-like kinetic equations and integro-differential equations (IDEs), typically cannot resolve individuals and, instead, track the ensemble average (EA) behaviour of a population. However, these models are more amenable to mathematical analysis and more readily provide insight into long-term behaviour than discrete frameworks do [42,43]. For example, changes in patterning may arise because of Turing-like instabilities [44–46] or due to alterations in physically based interactions such as cell–cell adhesion [7,38,47–50]. In the case of zebrafish patterns, researchers have applied a wide swath of continuous models—including reaction–diffusion equations [16,21,35,51,52] and non-local PDEs [7,53–55]—to better understand cell dynamics.

Despite the differences between discrete and continuous approaches, it is possible to establish a mathematical link between these representations in the limit of infinite individuals. This procedure, known as ‘coarse-graining’, derives differential equations from a given discrete model and yields information about its EA behaviour [56–62]. For example, the authors in [63–65] derive logistic IDEs from stochastic processes that describe the birth and death of individuals undergoing Darwinian evolution in the limit of large numbers. Coarse-grained descriptions become inaccurate when relatively few individuals are present, however, as is the case in many biological contexts such as pattern formation in zebrafish. Many approaches also neglect potentially important spatial correlations between cells—caused, for instance, by division or competition—that may play a critical role in pattern dynamics [52,66–71]. While it is possible to go beyond this ‘mean-field’ setting by deriving continuous models that respect higher-order correlations [72–76] and hard-core interactions [77], these methods still rely on simplifying assumptions that introduce errors between the discrete and continuous frameworks. Controlling these errors in a biological setting is an important objective.

We help tackle this problem by developing a pipeline to minimize spatio-temporal discrepancies between continuous models and individual-based data in settings with biologically relevant, dynamic cell numbers. The method relies on fitting parameters that effectively dilate the time variable of continuous-model solutions. Our approach can be used to describe biological self-organization in systems whose macroscopic description can be derived or inferred. We apply our approach to a case study motivated by stripe formation in zebrafish skin, as this allows us to illustrate its utility and interpretablity for experimentally measurable quantities with biologically meaningful spatial and temporal units. In §2, we describe the ABM that we simulate to generate synthetic individual-based data associated with self-organizing phenomena. In its full form, the ABM motivating our work [14] admits pattern formation via non-local rules for cell birth, death and movement that are inspired by the underlying biology of zebrafish-skin patterns (figure 1) [14,40]. To focus on the presentation of our pipeline, however, we simplify some biological complexity by reducing this model [14] to focus on a single cell type—melanophores or dense xanthophores. (We plan to extend this pipeline to multiple cell types in future work.) We then detail the corresponding continuous descriptions and our method for matching their solutions to EA ABM data even in scenarios with finite and time-dynamic cell numbers. We present our results for black melanophores in §3, and—as a means of demonstrating the generality of our methodology—apply the same approach to dense xanthophores in electronic supplementary material, S6.

## 2. Mathematical models and methods

In §2.1, we develop our ABM for cell migration and derive its continuous counterpart. Subsequently, in §2.2, we introduce our discrete model for cell birth and develop a corresponding continuous IDE model. We present our full models of migration and proliferation in §2.3. Lastly, we present our approach to estimating scaling parameters in our continuous models from EA ABM data in §2.4. Following previous ABMs [14,40] of pattern formation in zebrafish, we assume (i) migration is governed by conservative forces between pigment cells and (ii) non-local interactions inform cell birth in a two-dimensional (2D) plane [14,39,40]. Throughout this paper, we refer to:

$$\begin{array}{rl} \Omega \subset R^{2} & =\text{domain of the simulation with spatial units of millimetres (mm),}\\ R^{2}∋M_{i}(t) & =\text{coordinates of the centre of the }i\text{th}\text{ melanophore at }t\;\text{days in our discrete models,}\\ N∋N_{M}(t) & =\text{total number of melanophores present at time}\;t\;\text{days and}\\ R_{\geq 0}∋M(x,t) & =\text{density of melanophores at position}\;x\;\text{ and time}\;t\;\text{in}\;\mathrm{cells}\;{\mathrm{mm}}^{-2}, \end{array}$$

with the exception of figure 7 where we consider a one-dimensional (1D) domain; there *M*(**x**, *t*) is the number density of melanophores in cells mm^−1^. Because it appears several times, we define the indicator function $1_{\left\{\text{condition}\right\}}$ here as

2.1 $$1_{\left\{\text{condition}\right\}}(x)=\left\{ \begin{array}{cc} 1,\; & \text{if}\;x\;\text{satisfies the rule specified by }‘\text{condition'},\;\\ 0,\; & \text{otherwise, }\; \end{array}\right.$$

where ‘condition’ depends on the model rule and cell interaction, as we discuss next.

### 2.1. Models of migration

Our ABM for cell movement tracks the position, **M**_*i*_(*t*), of each cell, indexed by *i* ∈ {1, …, *N*_M_(*t*)}, at time *t* ≥ 0. The movement of each melanophore depends on its interactions with surrounding melanophores, following an overdamped version of Newton’s second law. The forces are assumed to be conservative, i.e. they may be written as the gradient of a potential. This leads to the following system:

2.2 $$\frac{\text{d}M_{i}}{\text{d}t}\approx F_{\text{int}}^{\left(i\right)}=-\sum_{\;j=1,j\neq i}^{N_{M}(t)}\nabla W_{\mathrm{MM}}^{c}(M_{i}-M_{j}).$$

Here, $F_{\text{int}}^{\left(i\right)}$ is the net force arising from all cell–cell interactions according to the potential $W_{\mathrm{MM}}(r)$, where **r** is the inter-particle displacement. The potential can encode cell–cell repulsion and adhesion, depending on the sign of its gradient along the direction between two cell centres. Many choices for $W_{\mathrm{MM}}(r)$ are possible, including harmonic, power-law, Morse and Lennard-Jones potentials, among others. Here, we use exponential potentials given by

2.3 $$W_{\mathrm{MM}}(r)=R_{\mathrm{MM}}\;e^{-|r|/\omega_{\mathrm{MM}}}-A_{\mathrm{MM}}\;e^{-|r|/a_{\mathrm{MM}}},$$

in accordance with prior ABMs of pattern formation in zebrafish [14,40]; see figure 1*c* and table 1 for parameter values and their biological interpretations. To model cells communicating through cellular extensions or dendrites [19,20], secreted signals [80] or cell–cell contact [81], we assume forces on cells are zero beyond some cut-off distance *d*_max_. We represent this using the notation $\nabla W_{\mathrm{MM}}^{c}(r)=\nabla W_{\mathrm{MM}}(r)\;1_{\left\{|r|<d_{\max}\right\}}(r)$, and set *N*_M_(*t*) = *N*_M_ when there is no cell birth. We remark that the model given by equation (2.2) is deterministic in the sense that it does not include Brownian motion.

**Table 1.** Model and simulation parameters used throughout the paper. We note that $N_{\mathrm{bir}}\in \{10,25,50,100,150,200,250\}$ realizations for 2D simulations and *N*_bir_ ∈ {1, 2, 3, 4, 5, 6, 7, 8, 9, 10} realizations for 1D simulations. As we discuss in the electronic supplementary material, S2, *N*_sim_ = 5 × 10^3^ realizations for most EA ABM solutions of the cell movement model and *N*_sim_ = 10^3^ for all EA ABM results of the cell birth and combined models. We set *N*_hist_ = 30 voxels for EA ABM solutions of the cell birth and combined models, and *N*_hist_ = *N*_bin_ for the cell movement model. (See figure captions for our *N*_bin_ values.) The values of $R_{\mathrm{MM}}$ (and $R_{\mathrm{XX}}$, see electronic supplementary material, S1.3) were reported as repulsion strengths (i.e. $R_{\mathrm{MM}}/\omega_{\mathrm{MM}}$) in [39].

parameter	value	description and motivation
$R_{\mathrm{MM}}$	0.00124 mm^2^ day^−1^	strength of melanophore repulsion potential in equation (2.3); based on [14,39]
*A*_MM_	0 mm^2^ day^−1^	strength of melanophore adhesion potential in equation (2.3); based on [14,39]
$\omega_{\mathrm{MM}}$	0.02 mm	melanophore repulsion interaction range in equation (2.3); based on [14,39]
*a*_MM_	0.012 mm	melanophore adhesion interaction range in equation (2.3); based on [14,39]
*d*_max_	0.2 mm	maximum cell interaction distance in equation (2.2); based on [14,39]
*d*_loc_	0.075 mm	maximum interaction range for cell birth in equation (2.6); based on [14] and chosen slightly larger than measurements of cell–cell distances [78,79]
*N*_bir_	varies	number of positions selected uniformly at random per day for possible cell proliferation (e.g. differentiation from precursors) in equations (2.5) and (2.7)
*c*^−^	1 cell	lower bound for the number of cells in a short-range neighbourhood for cell proliferation in equations (2.5) and (2.7)
*c*^+^	6 cells	upper bound for the number of cells in a short-range neighbourhood for birth in equations (2.5) and (2.7); based on estimations of data [16,78] in [14]
*t*_final_	150 or 2000 days	simulation end time (150 days in 2D and 2000 days in 1D)
Δ*t*_move_	0.01 or 0.1 days	time step for numerical implementation of equation (2.2) and electronic supplementary material, equation (S3)
Δ*t*_bir_	1 day	time step for numerical implementation of cell birth in equations (2.5) and (2.7)
Δ*t*_PDE_	0.05 days	time step for numerical implementation of equations (2.4), (2.8) and electronic supplementary material, equation (S6)
Δ*t*_record_	1 day	time step for recording data from model simulations
*N*_sim_	varies	number of ABM realizations for computing EA cell densities
$N_{\mathrm{bin}}$	varies	spatial discretization step for solving our continuous models
*N*_hist_	$N_{\mathrm{bin}}$ or 30 voxels	spatial discretization step for binning simulation results for comparison

**Figure 2.**
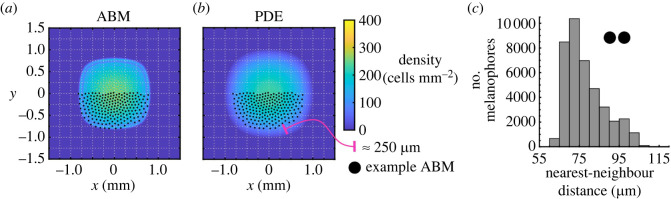
The PDE for cell migration does not accurately describe the EA ABM result when its scaling parameter, $\alpha_{\mathrm{MM}}$, is set to unity. (*a*) To compute our EA ABM result, we solve equation (2.2) using an initial condition of 400 melanophores placed uniformly at random in a 1 × 1 mm^2^ square, group cell positions in a 240 × 240 histogram, and average such data over 10^4^ ABM realizations. (*b*) We compute the corresponding PDE solution by simulating equation (2.4) with $\alpha_{\mathrm{MM}}=1$ from a uniform density of 400 cells mm^−2^ in the same square region. The ABM and PDE solutions use the same potential (given by equation (2.3) with parameters in table 1). We overlay an example ABM realization for comparison; the results demonstrate that the support of the PDE is larger than that of the ABM by about 200–250 μm. Because melanophore–melanophore distances have been measured to be roughly 50 μm *in vivo* [78] and stripes are only about 7–12 cells wide [16], this is a large difference. (*c*) The distribution of nearest-neighbour distances across 100 ABM realizations demonstrates that cell–cell separation ranges from roughly 60 to 100 μm. Based on visual inspection of the graphs, nearest-neighbour distances appear inversely proportional to the EA cell density. In (*a*–*c*), we show results at *t* = 150 days.

The associated continuous model describes the melanophore density, *M*(**x**, *t*). Integrating *M*(**x**, *t*) over a bounded region yields the total number of melanophores within that area at time *t*. Following the coarse-graining procedure in [60,82,83], an outline of which is found in electronic supplementary material, §1.1, we obtain the PDE below:

2.4 $$\frac{\partial M}{\partial t}=\alpha_{\mathrm{MM}}\nabla \cdot (M\nabla W_{\mathrm{MM}}^{c}⋆M),$$

where the force $\nabla W_{\mathrm{MM}}^{c}$ is the same as in equation (2.2) and ⋆ is the convolution operator [14,40]. The parameter $\alpha_{\mathrm{MM}}$ in equation (2.4) is not inherent to the coarse-graining procedure; rather, it accounts for possible differences between the discrete and continuous models. Indeed, simulating equation (2.4) with the parameter values listed in table 1 and $\alpha_{\mathrm{MM}}=1$ (the value expected from the mean-field approximation) does not always capture the ABM dynamics; see figure 2.

The individual and EA ABM results demonstrate that cells disperse until they are about 55–115 μm apart at *t* = 150 days. The PDE with $\alpha_{\mathrm{MM}}=1$, however, predicts that cells travel about 250 μm further in the same time period. Additionally, the PDE cell density is lower than the EA ABM density near the centre of the domain, implying that cells are more separated there. The continuous solution at earlier times more closely resembles the EA ABM result at *t* = 150 days, however, which suggests that PDE solutions evolve at a faster time scale than that of the discrete model. The parameter $\alpha_{\mathrm{MM}}$ effectively dilates the time variable, such that solutions travel $\alpha_{\mathrm{MM}}$ times more quickly. Thus, a non-unitary value of $\alpha_{\mathrm{MM}}$ is likely to produce a better match between the discrete and continuous solutions. To our knowledge, the value of $\alpha_{\mathrm{MM}}$ cannot be derived *a priori*. Instead, we develop an approach for estimating its value based on ABM data in §2.4, and will pursue an analytic derivation for this parameter in future work.

### 2.2. Models of cell birth

Our ABM for cell birth consists of stochastic, discrete-time rules which we adapt and simplify from [14] (motivation for these rules may be found in electronic supplementary material, S1.2). Specifically, at each time step (i.e. day) in a simulation, we select $N_{\mathrm{bir}}\in N$ locations uniformly at random from *Ω* and evaluate them synchronously for possible cell birth. Each selected location, **z**, represents the position of a precursor cell that may differentiate into a melanophore based on the signals that it receives. The conditions for melanophore birth in the ABM [14] depend on both neighbouring melanophores and dense xanthophores, as we show in electronic supplementary material, figure S1. Since we restrict to one population in this paper, we simplify the rules from [14]; see electronic supplementary material, S1.2, for details. In particular, a new melanophore emerges at position **z** according to the rule

2.5 $$\begin{array}{cc} & \underset{\text{short-range activation}}{\underbrace{\sum_{i=1}^{N_{M}}1_{\left\{M_{i}\in \Omega_{\mathrm{loc}}^{z}\right\}}(M_{i})\geq 1}}\;\text{and}\\ & \underset{\text{overcrowding prevention}}{\underbrace{\sum_{i=1}^{N_{M}}1_{\left\{M_{i}\in \Omega_{\mathrm{loc}}^{z}\right\}}(M_{i})<c_{\mathrm{ABM}}^{+}}}\;⟶\;\text{melanophore appears at }z, \end{array}\}$$

where $c_{\mathrm{ABM}}^{+}\in N$, and

2.6 $$\Omega_{\mathrm{loc}}^{z}=\text{disc centred at}\;z\text{ with radius}\;d_{\mathrm{loc}}.$$

According to equation (2.5), new cells appear near existing melanophores until the maximum number of cells—namely, $c_{\mathrm{ABM}}^{+}$—in $\Omega_{\mathrm{loc}}^{z}$, the interaction region between cells, is reached; see table 1 for parameter values. While equation (2.5) is deterministic, stochasticity enters our ABM through our *N*_bir_ randomly selected positions {**z**}. Similar stochastic rules can also be used to model cell death, as in [14,40], although we do not consider them here.

We do not know of existing methods for rigorously deriving continuous models of cell birth from off-lattice ABMs with this noise structure. We therefore adopt a phenomenological modelling approach, in which we create a continuous model whose governing equations mimic the stochastic interaction rules. We reason that the number density of cells in this setting must increase at a constant rate (proportional to *N*_bir_) when continuous versions of the overcrowding and short-range activation restrictions are met, since this occurs at the individual level in the ABM. Furthermore, we represent the density restrictions with an indicator function using the integral of the number density over Ω_loc_ as an argument, since the latter quantity yields the total number of cells within that region. This leads to the following model:

2.7 $$\frac{\partial M}{\partial t}(x,t)=\gamma N_{\mathrm{bir}}1_{\left\{1\leq \int_{\Omega_{\mathrm{loc}}^{x}}M(y,t)\;dy<c^{+}\right\}}(x,t),$$

where *c*^+^ is the continuous equivalent of the density-limiting parameter $c_{\mathrm{ABM}}^{+}$ in equation (2.5); *N*_bir_ has the same value as in our corresponding ABM; and $\gamma \in R^{+}$ is a parameter that effectively dilates the time variable in a similar way as $\alpha_{\mathrm{MM}}$ in the cell movement model. The units of *γ* must be inversely proportional to those of the domain size in order to make the dimensions of equation (2.7) consistent. Its value is unknown; however, we can employ a phenomenological argument to determine an expected value by integrating equation (2.7) over the whole domain. This yields an upper bound on the number of cells born per unit time of *γN*_bir_|*Ω*|, hence one expects *γ* ≈ |*Ω*|^−1^ to maintain a maximum rate of *N*_bir_ cells born per day as in our ABM. We note, however, that this argument does not take into account possible clustering or other spatial correlations that can occur in the discrete setting, which may change the values of *γ* and *c*^+^ from their expected values. While we could address this by allowing both parameters to depend on the proportion of the domain in which the birth conditions are fulfilled, we leave this extension for a future study and simply estimate uniform values for *γ* and *c*^+^ by fitting to EA ABM data, as we do for $\alpha_{\mathrm{MM}}$ in the movement-only model. We overview our approach for estimating the values of *c*^+^ and *γ* in §2.4.

### 2.3. Full models of cell movement and birth

We combine our descriptions of cell movement and proliferation to form our full discrete and continuous models. For our full ABM, we move cells according to equation (2.2) and then introduce new agents based on equation (2.5) at each simulated day; see electronic supplementary material, S2, for details. For our continuous model, we combine the terms related to movement and birth, such that the cell density evolves according to

2.8 $$\frac{\partial M}{\partial t}(x,t)=\alpha_{\mathrm{MM}}\nabla \cdot (M\nabla W_{\mathrm{MM}}^{c}⋆M)+\gamma N_{\mathrm{bir}}1_{\left\{1\leq \int_{\Omega_{\mathrm{loc}}}^{x}M(y,t)\;dy<c^{+}\right\}}(x,t),$$

where the parameters $\alpha_{\mathrm{MM}}$, *γ*, *N*_bir_ and *c*^+^ have the same interpretations as in §§2.1 and 2.2. Importantly, by assuming that these parameters have the same interpretations, we are assuming that migration and proliferation are additive, so that combining them has no extra influence. Our fitting approach for these parameters, discussed below, allows us to evaluate this choice and better understand the interplay of these two mechanisms in discrete and continuous settings.

### 2.4. Parameter estimation procedure

**Figure 3.**
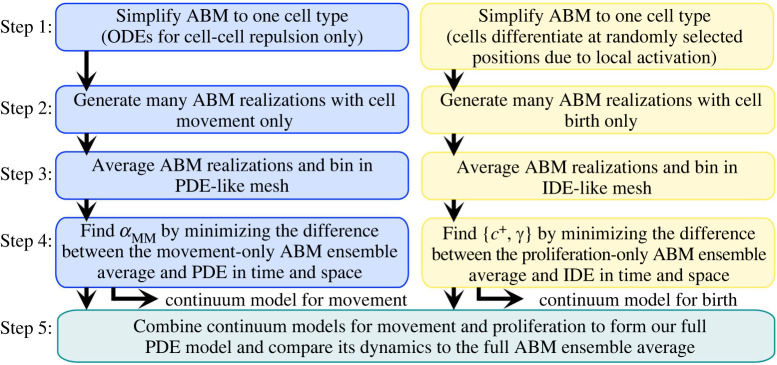
Our modular pipeline for matching the solutions of continuous and discrete models and identifying how cell movement and birth interact in both settings. We first isolate the discrete-model terms from [14] corresponding to movement (left column) and birth (right column) and simplify them to consider only one cell type. We then produce multiple realizations of our ABMs, sorting the cell locations into a grid of *N*_hist_ × *N*_hist_ voxels to yield the EA discrete-model results. We simulate our continuous model for cell movement (respectively, cell birth) and compare it on the same spatial mesh, with values of $\alpha_{\mathrm{MM}}$ (respectively, *c*^+^ and *γ*) obtained from a least-squares optimization approach; see electronic supplementary material, S2, for details. Finally, we combine the fitted movement and birth models to produce our full continuous model. While an extension of this pipeline to fit all three parameters simultaneously is straightforward, fitting separately allows us to better understand the effects of cell movement and birth in discrete and continuous frameworks.

**Figure 4.**
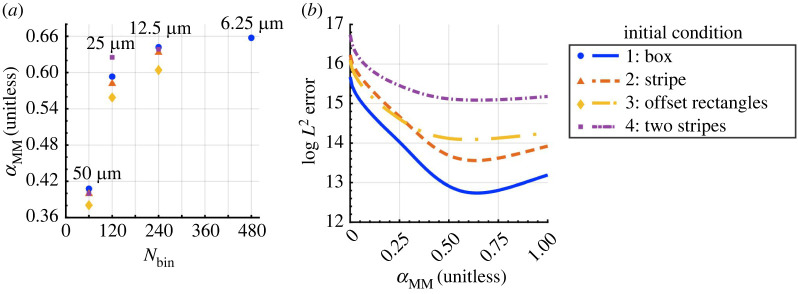
The optimal PDE scaling parameter for movement depends on the mesh resolution but appears to converge. (*a*) A scatter plot of the numerically optimized value of $\alpha_{\mathrm{MM}}$ from equation (2.4) as a function of the mesh resolution, $N_{\mathrm{bin}}$, demonstrates that this scaling parameter is correlated with the mesh resolution, but appears to converge at sufficiently high (i.e. $N_{\mathrm{bin}}\geq 240$) detail. (We omit 95% confidence intervals because these are so narrow that they are difficult to see.) (*b*) Plotting the $\log L^{2}$ error, given by equation (2.9), as a function of $\alpha_{\mathrm{MM}}$ with $N_{\mathrm{bin}}=240$ for each initial condition suggests that the values in (*a*) are optimal. As we show in figure 2*c*, melanophores are typically separated by more than 60 μm in our movement-only ABM simulations—over four times the voxel width in a grid with $N_{\mathrm{bin}}=240$. See §2.4 and electronic supplementary material, S2, for numerical details.

We identify the values of the parameters—$\alpha_{\mathrm{MM}}$ in equation (2.4) and {*γ*, *c*^+^} in equation (2.7)—by minimizing the sum of squared differences (hereafter referred to as the ‘*L*^2^ error’) between the continuous and EA discrete solutions over time and space. Because we are able to model the isolated processes of cell birth and movement separately, or consider them acting simultaneously, there are two ways of estimating parameters: by fitting all three parameters simultaneously to data from the combined model, or by fitting them in a modular fashion by considering cell movement and birth in isolation from each other. For the remainder of this paper, we adopt a modular approach because it allows us to probe the particular effects of cell movement and birth in detail (see figure 3 for an overview), and we present a study of simultaneous estimation in electronic supplementary material, S3. In particular, by using our modular parameter values in the combined PDE model, we can investigate their interplay and better understand the additive effects of individual-level mechanisms on the accuracy of continuous models. As we discuss in electronic supplementary material, S3, our modular approach may also supply additional information that can improve parameter estimation. For example, we show in electronic supplementary material, S3, that $\alpha_{\mathrm{MM}}$ and *γ* are not uniquely identifiable if they are fit only to the combined EA ABM data, whereas figure 4*b* and figure 7*f* suggest we can uniquely identify them with a modular approach. We overview our method for parameter estimation below; for parameter values, see table 1. We refer to electronic supplementary material, S2–S3, for further details about our implementation of the pipeline in addition to alternative choices that could be taken in parameter estimation (such as fitting to earlier times, less refined spatial data etc.).

We consider biologically meaningful time scales (i.e. days), length scales (i.e. mm), and cell densities and stress empirical units throughout our results. This choice supports future studies that may treat pattern formation with multiple cell types. Throughout our simulations, we consider a domain of size 3 × 3 mm^2^ (with one 1D exception in figure 7). We implement four initial conditions to extract common features of cell interactions from different geometric scenarios. The first involves a square region of melanophores in the centre of the domain (*Box*), which mimics the symmetry of the domain. For the second initial condition, we place a single stripe of melanophores (*Stripe*), which is motivated by the typical patterning observed in wild-type zebrafish. For the third initial condition, we consider two rectangular regions of melanophores (*Offset rectangles*), which take into account non-standard geometries and the meeting of two disjoint melanophore populations. Finally, the last initial condition we consider involves two melanophore stripes (*Two stripes*), which explores the interactions between two disjoint melanophore populations with biologically realistic sharp fronts. (See electronic supplementary material, figure S6, for a summary of these initial conditions.) We initialize individual ABM simulations by sampling cell positions uniformly in these regions for each respective initial condition, and initialize our continuous models by setting the cell density uniformly equal to the estimated biological density of 400 cells mm^−2^ [14].

In Step 2 of our pipeline in figure 3, we solve our discrete models with an explicit approach. Specifically, we solve equation (2.2) with an explicit forward Euler scheme. To model differentiation from uniformly distributed precursor cells [14], we solve the birth-only ABM by selecting *N*_bir_ sites in the domain uniformly at random at a fixed time step (here, 1 day) and placing a new cell at each position that meets the conditions given by equation (2.5). (Following the approach in [14,39,40], we evaluate all *N*_bir_ locations for potential cell proliferation at the same time. This synchronous evaluation means that it is possible, though uncommon, for more than $c_{\mathrm{ABM}}^{+}$ cells to be present in a local neighbourhood, and the choice of parameters in our model, based on the ABMs [14,39,40], accounts for this possibility.) We solve our ABM combining migration and birth by simulating equation (2.2) and then implementing cell birth as above, with migration evaluated using a shorter time step than the time step for birth events.

To compare ABM results directly with the cell density from our continuous models, we obtain an EA distribution by simulating many ABM realizations (here, between 10^3^ and 10^4^ simulations), sorting all the cell locations into a histogram of *N*_hist_ × *N*_hist_ voxels (or *N*_hist_ × 1 voxels in 1D), and normalizing by the number of simulations and the voxel area for each day simulated; see Step 3 of our pipeline in figure 3. Other ways of relating ABM and PDE results are also possible, for example by introducing Gaussian kernels at each cell location [84]. However, as far as the construction of the histogram is concerned, we expect comparable results for particles and localized Gaussian kernels. Furthermore, we show in electronic supplementary material, S3, that histogram voxel size used to bin EA ABM data and the final time used to fit the continuous equations only play a minor role in affecting the parameter values we obtain, at least for the cell movement model.

As part of Step 4 of our pipeline in figure 3, we need to solve our continuous models, and we do so with explicit approaches. Specifically, we apply a first-order finite volume scheme for the migration model (equation (2.4)), a forward Euler method for the continuous cell birth model (equation (2.7)), and a combined finite volume/forward Euler scheme for the full continuous framework (equation (2.8)). (More details about the particular time steps used to simulate the discrete and continuous models are in electronic supplementary material, §2.) We simulate the continuous models on an $N_{\mathrm{bin}}\times N_{\mathrm{bin}}$ mesh and, to match with EA ABM solutions, record the average cell density at each day on a (possibly coarser) grid of *N*_hist_ × *N*_hist_ voxels.

We compute continuous model parameters by minimizing the *L*^2^ error between the continuous and EA ABM results across time. Notably, this nonlinear least-squares problem is equivalent to maximum likelihood parameter estimation when the densities produced from the ABM simulations are independent, identically distributed normal random variables with constant variance and mean equal to the continuous solution. The *L*^2^ error that we minimize is given by

2.9 $$\begin{array}{rl} e_{L^{2}}^{2}=\|M_{\mathrm{cts}}-M_{\mathrm{ABM}}{\|}_{L^{2}}^{2} & =\int_{t=0}^{t=t_{\mathrm{final}}}\int_{\Omega }{(M_{\mathrm{cts}}(x,t)-M_{\mathrm{ABM}}(x,t))}^{2}\;dx\;dt\\ & \approx \Delta t_{\mathrm{record}}\Delta x\Delta y\sum_{n=0}^{N_{T}}\sum_{i=1}^{N_{\mathrm{hist}}}\sum_{\;j=1}^{N_{\mathrm{hist}}}{\left(M_{\mathrm{cts},i,j}^{\left(n\right)}-M_{\mathrm{ABM},i,j}^{\left(n\right)}\right)}^{2}, \end{array}$$

where Δ*t*_record_ denotes the time steps at which data are collected; Δ*x* and Δ*y* are the spatial step sizes of the histogram used to compare the EA ABM and PDE data; $M_{\mathrm{cts},i,j}^{\left(n\right)}$ is the continuous-model solution at time *t*_*n*_ and position (*x*_*i*_, *y*_*j*_); and $M_{\mathrm{ABM},i,j}^{\left(n\right)}$ is the corresponding EA ABM result. For the birth-only model, we consider fitting to either the *L*^2^ error as above or simply the difference in the total cell count of the two datasets (we verify in electronic supplementary material, S2, table S4, that fitting to the *L*^2^ error produces similar parameter estimates). When we consider cell birth, we simulate our models with different values of *N*_bir_ and estimate parameters by minimizing the sum of the errors across these *N*_bir_ values. We fit parameters related to cell proliferation sequentially—that is, we determine the optimal value for *c*^+^ before *γ*. We verify in 1D that sequential and simultaneous estimation does not lead to significant difference in parameter values; see electronic supplementary material, S3.

## 3. Results

We now present our results linking discrete and continuous models of cell migration (§2.1), birth (§2.2), and migration and birth (§2.3). We first isolate each interaction process, separately identifying the values of $\alpha_{\mathrm{MM}}$ in equation (2.4) and {*γ*, *c*^+^} in equation (2.7). As we note in §2.4, this choice allows us to extract the distinct effects of each mechanism. We then determine how this simplification affects the ability of the full continuous framework, given by equation (2.8), to approximate EA ABM solutions. By considering different initial conditions (we discuss the details and motivations for these in electronic supplementary material, S4), we demonstrate the robustness of our fitting procedure. Our results show how the time scales of proliferation and movement in our continuous model may depend on numerical implementation and the frequency of stochastic cell birth controlled by *N*_bir_. Moreover, our modular fitting approach highlights important considerations to account for in more general systems where agents are moving and changing in number.

### 3.1. Cell migration

We estimate $\alpha_{\mathrm{MM}}$, the scaling parameter that controls the dynamics of melanophore movement. Figure 4*a* presents the values of $\alpha_{\mathrm{MM}}$ that minimize the *L*^2^ error between the continuous solution of equation (2.4) and EA ABM results for our four initial conditions (see §2.4 and electronic supplementary material, S2–S4). In each case, the optimal value of $\alpha_{\mathrm{MM}}$ is positively correlated with our PDE mesh resolution, i.e. greater values of $\alpha_{\mathrm{MM}}$ are associated with larger $N_{\mathrm{bin}}=N_{\mathrm{hist}}$ values. This unitless parameter appears to converge to around 0.60–0.66 as the mesh resolution increases. There is at most a 2.5% relative difference between the values of $\alpha_{\mathrm{MM}}$ that we find when $N_{\mathrm{bin}}=240$ versus when $N_{\mathrm{bin}}=480$ for our *Box* initial condition. These results suggest that $\alpha_{\mathrm{MM}}$ is independent of the mesh resolution when the latter contains at least 240 × 240 voxels, corresponding to a mesh spacing of 12.5 μm. As we show in figure 2*c*, melanophores tend to separate by between 60 and 100 μm in our ABM results, so this mesh spacing is less than one quarter of the typical distance between agents.

At each mesh resolution in figure 4*a*, the estimated optimal value of $\alpha_{\mathrm{MM}}$ does not appear to depend greatly on the initial condition. For example, in the case of a mesh with $N_{\mathrm{bin}}=N_{\mathrm{hist}}=240$, the maximum relative difference between the four parameter values is at most 6.5%. This similarity suggests that there is an inherent time scale at which migratory melanophore–melanophore interactions occur. Figure 4*b*, which presents the $\log L^{2}$ error for $N_{\mathrm{bin}}=240$ as a function of $\alpha_{\mathrm{MM}}$, further supports this conclusion. Although the errors associated with different initial conditions can vary by an order of magnitude, the minimum value of each (roughly convex) curve appears nearly identical and is located near the values shown in figure 4*a*.

**Figure 5.**
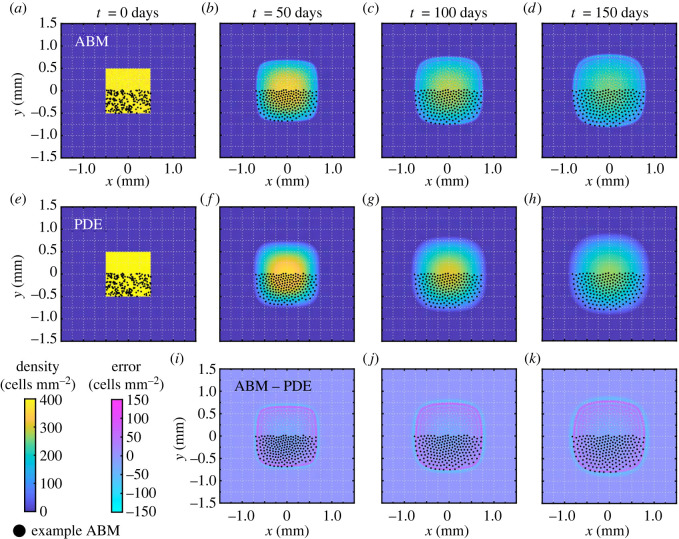
Melanophore movement models with our *Box* initial condition. We present (*a*–*d*) snapshots of the EA cell density (cells mm^−2^) across 10^4^ ABM realizations, (*e*–*h*) the corresponding PDE results using the optimal value of $\alpha_{\mathrm{MM}}$ for a mesh resolution of $N_{\mathrm{bin}}=240$, and (*i*–*k*) the error between the PDE and EA ABM densities. We overlay cell positions for one example ABM simulation (black points) as a visual guide; we show roughly half of the region occupied by these cells. A difference in cell density of 150 cells mm^−2^ in a given voxel corresponds to about 0.0234 cells for this choice of mesh resolution. We find that the average pointwise errors (over voxels where at least one of the EA ABM or PDE solutions is non-zero) are about 28 cells mm^−2^, 17 cells mm^−2^, 17 cells mm^−2^ and 17 cells mm^−2^ at *t* = 0, 50, 100 and 150 days, respectively (these values correspond, respectively, to roughly 7%, 4%, 4% and 4% of the maximum cell density of 400 cells mm^−2^).

Figure 5 presents snapshots of the EA ABM results across 10^4^ realizations of equation (2.2) and the optimized PDE solution associated with the *Box* initial condition. The first row shows the expansion in time of the EA ABM support, i.e. the area occupied by the cells, due to melanophore–melanophore repulsion. For more intuition, we superimpose the cell positions from one ABM realization on our number-density results in this figure and throughout the paper. In all cases, we crop out approximately the upper half of cell positions. Visual inspection of cell positions in figure 5 suggests that melanophore–melanophore distances increase near the edge of the collective. Similarly, the speed at which the support expands appears to slow down for the EA ABM result, consistent with melanophores experiencing weaker forces from comparatively distant cells in this region.

We also observe in figure 5*a*–*d* that a band of high cell density emerges around the edge of the support which surrounds a ring-like region of low density. These bands may result from the combined effects of cell–cell repulsion and the fine mesh resolution that we use to sort agent positions in the EA solution. Repulsion causes cells at the edge of the collective to travel towards empty regions, while more centrally located agents move more slowly due to the balance of forces from their neighbours. When repulsion separates cells by distances greater than the mesh resolution, we expect regions of low density within the solution support to appear. These oscillatory bands should become less evident when the repulsive potentials in figure 1*d* exhibit shallower gradients, as this permits cells to cluster more closely, or when coarser histograms with fewer bins are used to visualize the EA ABM data. As we discuss in electronic supplementary material, S6, the forces acting on xanthophores are about an order of magnitude smaller than those for melanophores, and we indeed observe less pronounced bands there. Notably, fitting to EA ABM data on coarser histograms leads to similar parameter estimates; see electronic supplementary material, S3, for details.

We present snapshots of the continuous model, equation (2.4), under our estimated value of $\alpha_{\mathrm{MM}}$ in figure 5*e*–*h*. This PDE solution captures the dynamics of our example ABM realization significantly better than the case in figure 2*b*, when $\alpha_{\mathrm{MM}}=1$. However, unlike the EA ABM result, the PDE does not exhibit bands of high and low cell density. This discrepancy can be further appreciated in figure 5*i*–*k*, which presents snapshots of the pointwise difference between the PDE and EA ABM solutions. Here, positive values indicate that the discrete solution is larger than the continuous one. The lack of bands in the PDE setting is likely because the mean-field assumption used to derive the continuous system is invalid where density is low. We do not expect this discrepancy to be as pronounced in models that include cell birth, as this mechanism increases density; see §3.3. Moreover, the PDE support expands more quickly than that of the ABM. This result is likely due to our choice of error function to fit $\alpha_{\mathrm{MM}}$. Specifically, this parameter is biased towards values that produce accurate approximations in the bulk as these regions have a larger contribution to the *L*^2^ norm. Since we have already determined that the assumptions underlying the continuous model break down in low density regions, however, we choose to fit to the bulk of the cell density and focus on the *L*^2^ difference.

**Figure 6.**
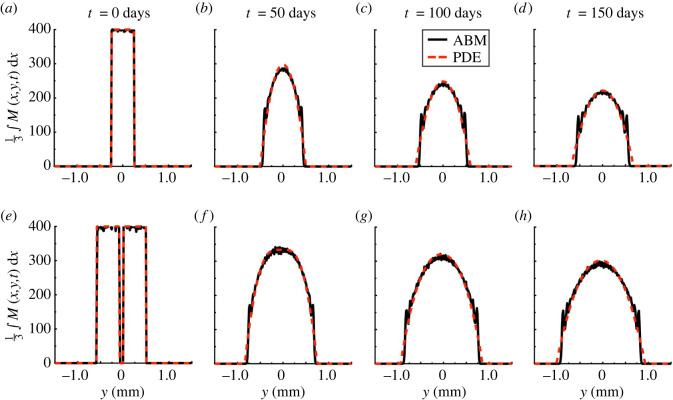
Melanophore movement models with our *Stripe* and *Two stripes* initial conditions. (*a*–*d*) We present snapshots of the column-averaged cell density (cells mm^−2^, black solid line), generated from 10^3^ ABM realizations for the *Stripe* case, alongside the corresponding PDE solution (dashed red line) under a mesh resolution of $N_{\mathrm{bin}}=240$ and our optimized value of $\alpha_{\mathrm{MM}}$. (*e*–*h*) Similarly, we show snapshots of the column-averaged density, generated from 10^3^ ABM realizations for the *Two stripes* case, and the corresponding PDE solution. The 2D solutions are nearly uniform in the *x*-direction (data not shown).

To demonstrate that our observations for the *Box* case are consistent across initial conditions, we compare the EA ABM and PDE dynamics for the *Stripe* and *Two stripes* initial conditions in figure 6; electronic supplementary material, figure S7, presents results for the *Offset rectangles* initial condition. In figure 6*a*–*d*, the column-averaged PDE solution, i.e. the solution average over the *x* variable, has a larger support than that of the EA ABM and does not exhibit oscillatory bands. (Comparing column averages is justified because both results are nearly uniform along the *x*-axis.) Nevertheless, the continuous solution closely approximates the EA ABM density, particularly in regions where the latter is high. For example, we find that the average pointwise error (over voxels where at least one of the EA ABM or PDE solution is non-zero) is equal to about 40 cells mm^−2^ at *t* = 0 days, 24 cells mm^−2^ at *t* = 50 days, 23 cells mm^−2^ at *t* = 100 days and 23 cells mm^−2^ at *t* = 150 days (these correspond to roughly 10%, 6%, 6% and 6% of the maximum cell density of 400 cells mm^−2^, respectively). Both solutions invade empty space in time, and the speed of this travelling wavefront appears to slow as cells become more diffuse. For the *Two stripes* initial condition in figure 6*e*–*h*, the ABM and PDE predict that cells move into the initially empty space between stripes to approach a characteristic profile also observed in the one-stripe case. The EA ABM model does not appear to form oscillatory bands in the interstripe region, corroborating our hypothesis that these bands are more likely to arise near the edge of the solution support. In this case, the average pointwise error between the continuous and discrete data is roughly 18%, 11%, 11% and 11% of the maximum cell density at *t* = 0, 50, 100 and 150 days, respectively.

### 3.2. Cell birth

**Figure 7.**
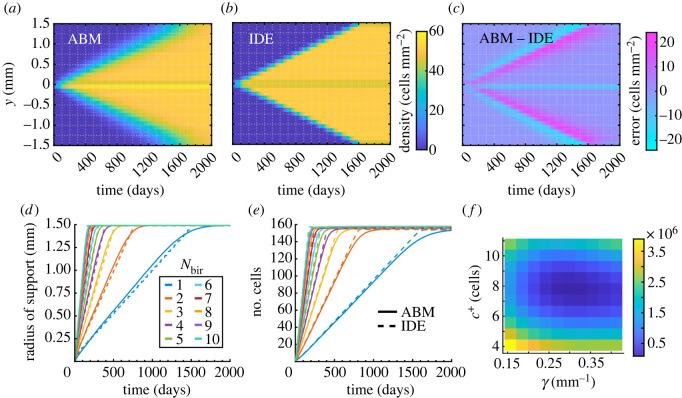
Melanophore birth models with a baseline initial condition of one cell at *y* = 0 in a 1D domain. Results in (*a*–*c*) are for *N*_bir_ = 1 positions day^−1^. (*a*) We compute the EA ABM result by simulating 10^3^ realizations of our ABM birth model, equation (2.5), and binning cell positions in a histogram with 0.1 mm-wide voxels (i.e. *N*_hist_ = 30). (*b*) We use a finer mesh resolution to solve our corresponding IDE model (equation (2.7)) before transferring results to the same histogram in (*a*) to perform parameter estimation. Here, we show our IDE solution produced with optimal parameter values *c*^+^ = 7.592 cells and *γ* = 0.2822. (*c*) The difference between our discrete and continuous results highlights that the ABM support is wider than the PDE support. (*d*) This is also visible by comparing their mean radii of support in time. To compute the mean radius of support for the ABM at a given time, we find the most distant melanophore from *y* = 0 for each simulation and average across these values. (*e*) Cell mass grows linearly in both models at first, but stochastic effects coupled with our overcrowding condition drive down the growth rate of the ABM as the domain fills with cells. (*f*) Plotting the squared *L*^2^ space–time difference between the discrete and continuous densities, summed over all *N*_bir_ values considered (namely *N*_bir_ = 1, …, 10), as a function of the density-limiting parameter *c*^+^ and birth-rate scaling parameter *γ* highlights its convex shape in *c*^+^ and lesser sensitivity to *γ*. We compute this *L*^2^ difference using a time step of 10 days here, and our results are based on 10^3^ simulations for each *N*_bir_ value; see electronic supplementary material, S3, for parameter values under alternative choices in our estimation process.

We identify the density-limiting parameter *c*^+^ and growth rate *γ* in our IDE model, equation (2.7), by comparing with agent-based data from equation (2.5). Importantly, the dynamics of discrete-model proliferation, unlike cell migration, involve stochasticity beyond the initial condition. To gain intuition, we thus start with 1D simulations: for each value of *N*_bir_ ∈ {1, 2, …, 10}, we compute the EA of 10^3^ ABM realizations from an initial condition in which a single melanophore is placed at the origin in a 1D domain. In figure 7*a*, we show the EA result for *N*_bir_ = 1 and the corresponding IDE model solution with the optimal values of *γ* and *c*^+^ in figure 7*b*. The continuous solution appears to have a smaller radius of support than the EA ABM result at every time point; see figure 7*c*. This result holds across all *N*_bir_ values in figure 7*d*. While the IDE predicts a piecewise linear growth of the total number of cells, the corresponding EA ABM result increases linearly before slowly saturating as the domain fills, as we depict in figure 7*e*. This behaviour likely arises from our overcrowding condition that prevents cell densities from exceeding *c*^+^. As the domain fills with cells, it becomes less likely to select a location **z** that satisfies the overcrowding condition in the ABM. This reduces the population growth rate at later times. By contrast, the IDE model specifies that the support increases by the same amount at each time step until it reaches the domain boundaries. As we discuss in §4, capturing discrete model behaviour more accurately at higher cell numbers may require replacing *γ* in our IDE with a density-dependent function.

Our 1D simulations provide a baseline case to test our estimation process. As we note in §2.4, we employ a sequential procedure, first fitting *c*^+^ with *γ* = |Ω|^−1^ and then estimating *γ* with *c*^+^ fixed. In the 1D case, this leads to optimal values *c*^+^ = 7.592 cells and *γ* = 0.2822. If we instead estimate both parameters simultaneously, we find *c*^+^ = 7.430 cells and *γ* = 0.2902. This is a difference of about 2.1% in *c*^+^ and 2.8% in *γ*, suggesting that sequential estimation reduces computational complexity without strongly affecting parameter values. To understand if a coarser discrepancy measure based only on cell numbers at each time is sufficient, we also fit *c*^+^ and *γ* by minimizing the squared difference in the total cell numbers over time; see electronic supplementary material, S2 and table S4, for the resulting parameter values. The corresponding parameter estimates differ from the density-based case by approximately 1.2% for and *c*^+^ and *γ*, suggesting both error measures are reasonable. Both approaches also appear to exhibit similar sensitivity as parameters are varied (compare figure 7; electronic supplementary material, figure S2).

**Figure 8.**
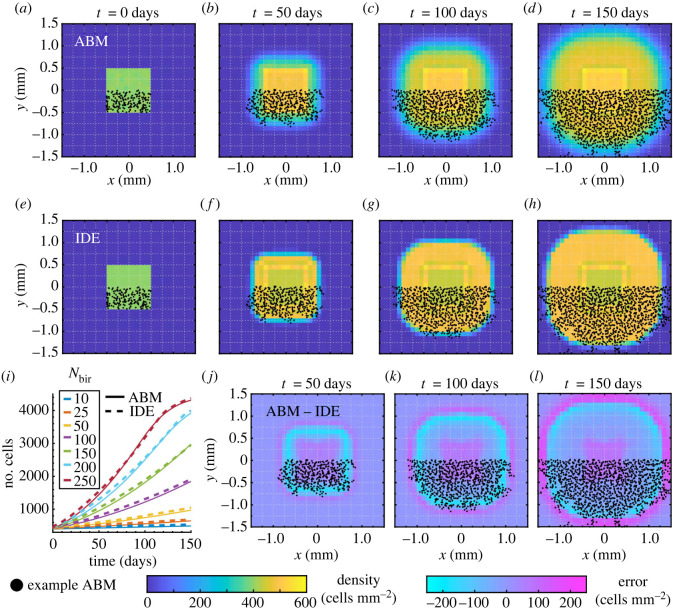
Melanophore proliferation models with our *Box* initial condition. Results in (*a*–*h*) and (*j*–*l*) are for *N*_bir_ = 150 positions day^−1^. We compute (*a*–*d*) the EA ABM result across 10^3^ simulations, and (*e*–*h*) the solution of our IDE model with optimal parameters *c*^+^ = 8.564 and *γ* = 0.1274. (*i*) Our continuous model captures the mean number of cells in our ABM simulations for different *N*_bir_ values well across time. (*j*–*l*) As in the 1D case in figure 7, the difference between the IDE and EA ABM results demonstrates that the ABM support extends beyond the IDE support. To provide more intuition, we overlay roughly half of the cell positions from an example ABM simulation in (*a*–*h*) and (*j*–*l*). See electronic supplementary material, figure S8 and table S4, for corresponding simulations using our *Offset rectangles* initial condition.

Figure 8 and electronic supplementary material, figure S8, respectively, show that proliferation in 2D broadens the solution support from the *Box* and *Offset rectangles* initial conditions over time, and the IDE model accurately captures the total cell mass of the ABM system for all *N*_bir_ values considered. Our estimated optimal values of *c*^+^ and *γ* for these two initial conditions differ by about 2.5% and 0.31%, respectively, suggesting that our estimation procedure is robust to the initial condition. We also highlight that a region of higher density forms at the edge of the initial condition’s support for both the ABM and IDE in figure 8*a*–*h*. Indeed, if **z** is near the support boundary, $\Omega_{\mathrm{loc}}^{z}$ covers only a fraction of the occupied domain, thereby meeting both conditions for birth. Conversely, the cell density at the centre of the domain is comparatively low throughout time because the total number of cells contained within discs of size $\left|\Omega_{\mathrm{loc}}\right|$ is already close to the threshold *c*^+^. Interestingly, as in the 1D case with only proliferation, the ABM EA support is larger than that of the IDE solution, the reverse of the behaviour that we observed for cell migration in figure 5.

### 3.3. Cell movement and proliferation

**Figure 9.**
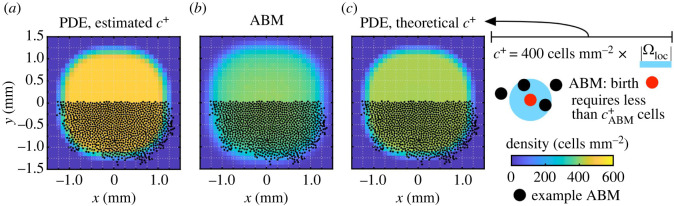
A modular approach to fitting parameters for cell movement and birth does not account for the interplay between these two mechanisms. We show results in (*a*–*c*) at *t* = 100 days for *N*_bir_ = 150 positions day^−1^. (*a*) The solution of our full PDE model (equation (2.8)) with the values of $\alpha_{\mathrm{MM}}$ and {*c*^+^, *γ*} that we fit based on ABM simulations of cell movement and birth, respectively, captures the support of the ABM EA result, but not its density. (*b*) In comparison, the density for the full discrete model is roughly 400 cells mm^−2^. (*c*) By integrating this density, which is based on empirical estimates of melanophore–melanophore distances [14,78], over an Ω_loc_-region, we find that *c*^+^ ≈ 7.0686 cells. With this value of *c*^+^, alongside the values of $\alpha_{\mathrm{MM}}$ and *γ* that we estimated for migration and birth individually, our PDE produces cell densities that more accurately represent the ABM dynamics.

To obtain a full continuous model, we may substitute our estimated values of the migration scaling parameter $\alpha_{\mathrm{MM}}$, density-limiting parameter *c*^+^ and birth-rate scaling parameter *γ* into equation (2.8). However, comparing this model to the dynamics of our full ABM shows that migration and proliferation have interwoven effects. To illustrate this phenomenon, we present a PDE solution with our optimal values of $\alpha_{\mathrm{MM}}$, *c*^+^ and *γ* from §§3.1 and 3.2 at *t* = 70 days in figure 9*a*. We observe that this PDE model produces a significantly higher cell density than its discrete counterpart in figure 9*b*. This discrepancy occurs regardless of the value of *N*_bir_, which influences the speed of cell birth. Related to this, we note that the long-time cell density in our ABM results is much lower when both mechanisms operate simultaneously than it is when only birth occurs; compare figure 8*d* and figure 9*b*. On the other hand, the inclusion of movement does not influence the long-time density of the continuous model solution; see the colourbar in figure 8*h* in comparison to the one in figure 9. Although we do not furnish these observations with an analytical explanation here, they demonstrate an interesting difference in how ‘adding’ mechanisms or terms impact PDE and ABM dynamics.

**Figure 10.**
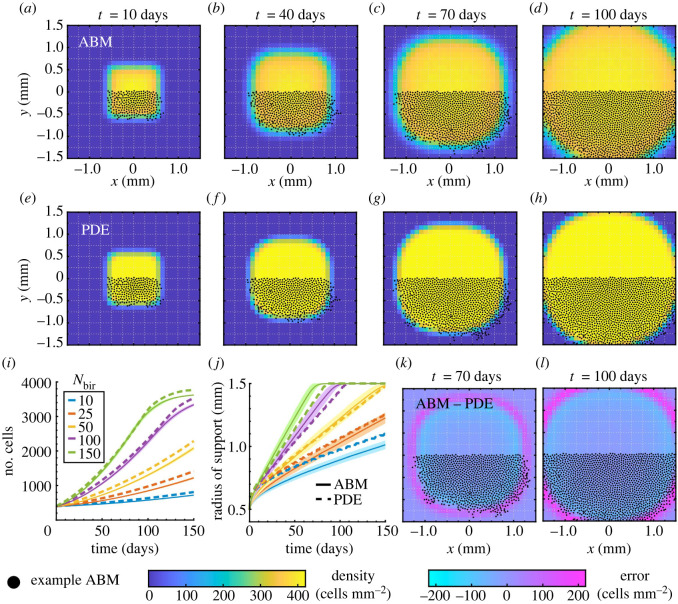
Melanophore movement and birth models with our *Box* initial condition. Results in (*a*–*h*) and (*j*–*l*) are for *N*_bir_ = 150 positions day^−1^. We (*a*–*d*) compute the EA ABM result using 10^3^ simulations, and (*e*–*h*) generate the PDE solution of equation (2.8) with *c*^+^ = 7.0686 cells and the values of $\alpha_{\mathrm{MM}}$ and *γ* that we estimated in §§3.1 and 3.2, respectively. (*i*) The time evolution of the PDE cell mass agrees well with the mean number of cells for the ABM under different *N*_bir_ values. (*j*) Depending on the time scales of migration and birth, the approximate PDE radius of support overtakes or trails the corresponding EA ABM result. We compute the radius of support for each ABM realization by finding the most distant cell from the origin at each time step; we then average these values across our simulations. In the PDE case, we find the furthest voxel with non-zero density from the origin based on the *L*^∞^ distance, after setting the density to zero if it is below single-digit precision of 10^−7^. (*k*,*l*) We show the difference between the PDE and EA ABM solutions from (*a*–*h*) at two sample times. We overlay cell positions from one ABM simulation to illustrate how the continuous and discrete solutions are related. In (*i*,*j*), shaded regions denote plus or minus one standard deviation of the EA ABM solution.

**Figure 11.**
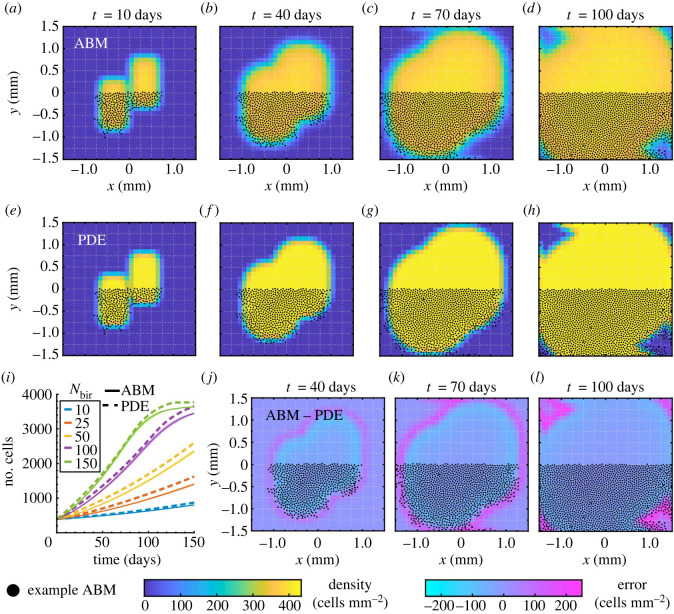
Melanophore movement and birth models with our *Offset rectangles* initial condition to verify that our fitting procedure is robust in non-standard geometries. Results in (*a*–*h*) and (*j*–*l*) are for *N*_bir_ = 150 positions day^−1^. As in figure 10, we show (*a*–*d*) the EA ABM result across 10^3^ simulations; (*e*–*h*) the corresponding PDE solution with *c*^+^ = 7.0686 cells and the values of $\alpha_{\mathrm{MM}}$ and *γ* that we estimated in §§3.1 and 3.2, respectively; (*i*) the PDE cell mass in time compared to the mean number of cells across 10^3^ ABM simulations for different *N*_bir_ values; and (*j*–*l*) the difference between the PDE and EA ABM solutions from (*a*–*h*). To provide more intuition, we overlay some cell positions from one ABM simulation.

One approach to addressing these discrepancies is to refit all three scaling parameters ($\alpha_{\mathrm{MM}}$, *γ* and *c*^+^) simultaneously, and we present the results of this approach in electronic supplementary material, S3. (Indeed, we show there that the errors produced with a simultaneous estimation approach can be relatively small, although the model parameters may not all be identifiable.) Because we are interested in understanding the interplay of individual-based mechanisms of proliferation and movement in continuous models, however, we instead take a simpler theoretical approach. Namely, we note that the parameter *c*^+^ is largely responsible for controlling the maximum cell density over long time periods. (We determine this by integrating equation (2.8) over space and identifying the steady-state dynamics; this analysis reveals that equilibrium is reached when the density within any neighbourhood $\Omega_{\mathrm{loc}}^{x}$ is below *c*^+^.) In order to limit the maximum density to our estimated empirical value of 400 cells mm^−2^ [14,78], we let *c*^+^ = 400|Ω_loc_| ≈ 7.0686 cells. As we show in figure 9*c*, using this value of *c*^+^, alongside our previously fitted values of $\alpha_{\mathrm{MM}}$ and *γ*, produces PDE densities that are much closer to the corresponding ABM results. We thus fix *c*^+^ = 7.0686 cells for the remainder of this paper, which allows us to highlight the time dynamics of our full PDE model in comparison to the EA ABM result with *Box* and *Offset rectangles* initial conditions in figures 10 and 11, respectively.

Figure 10*j*, which depicts the time evolution of the estimated radius of support for the PDE and EA ABM results, shows that a reasonably accurate continuous description of the combined model can be obtained by using the scaling parameters obtained from a modular approach. The supports of the ABM and PDE solutions both increase at roughly the same rate, although the degree to which the solutions agree can be affected by *N*_bir_: when this parameter is small, the PDE solution travels at a faster rate than the ABM solution, whereas the opposite occurs when this value is large (greater than *N*_bir_ = 50 positions day^−1^). At intermediate values of *N*_bir_ (i.e. *N*_bir_ = 50 positions day^−1^), however, the ABM and PDE solution curves are almost identical. Figure 10*i*, which presents the number of cells over time, yields similar observations: the ABM and PDE solutions exhibit similar dynamics over the time period investigated here and there are certain values of *N*_bir_ for which the solution curves are nearly identical. Figure 11 further demonstrates that these observations do not depend on the specific choice of initial condition. Figures 10 and 11 demonstrate that combining movement with proliferation also dissipates the oscillatory bands that we observed for movement alone in figure 5. This is likely because the stochastic addition of cells in the birth model disrupts the regular cell spacing created by the movement model. Furthermore, the EA ABM and PDE solutions exhibit similar characteristic profiles without regions of high cell density around the edge of the initial condition support, in contrast to the birth-only model (figure 8).

## 4. Discussion

We presented a procedure for constructing experimentally interpretable continuous models of cell migration and birth in biologically relevant settings of low numbers of individuals and localized interactions which may lie outside the validity of the mean-field regime. Specifically, we introduced and estimated scaling parameters in continuous models to account for realistic—i.e. relatively small and changing—numbers of cells with localized interactions. We applied this methodology to an illustrative, simplified example inspired by zebrafish pattern formation, in which we used a reduced ABM to generate individual-level data with biologically meaningful spatial and temporal units. Non-local rules for cell birth and migration, based on the ABM [14], informed our discrete and continuous descriptions and allowed us to transfer biological length scales and units to the macroscopic setting. Throughout our work, we stressed matching the spatio-temporal behaviour of our continuous and discrete models. We adopted a modular approach by estimating parameters in cases with either movement or birth before considering both mechanisms simultaneously. This allowed us to examine the specific contributions of each mechanism to self-organization and provided insight into their interplay in discrete and continuous settings.

We observed that the solutions of our continuous models expand at a different rate than EA ABM results and feature smoother profiles. Indeed, inaccuracies in mean-field descriptions for ‘intermediate’ numbers of individuals appear to be common in other biological phenomena described by simpler dynamics such as Fisher-KPP-type equations, cf. [85,86]. In fact, both references analytically derive corrections to the wave speed, a procedure we cannot adopt due to our use of off-lattice models. However, this substantiates our introduction of scaling parameters to handle the discrepancy. By introducing and estimating parameters that rescale the time variable, we produced more accurate descriptions of agent-based movement or birth. However, when we used the same parameter values in a continuous model of both cell migration and birth in §3.3, the PDE did not produce close estimates of the full ABM. Specifically, our full continuous model yielded larger long-time densities than the EA ABM results, motivating us to re-estimate the threshold value *c*^+^ with a theoretical approach. This generated a more faithful continuous description and highlighted that the effects of movement and proliferation are not simply additive. We thus stress that parameters must be fitted to data in which all mechanisms of interest act simultaneously, in order to capture their interplay. This is particularly crucial for contexts such as cancer biology, where cell migration, proliferation and death are known to play critical roles in tumour progression and immune response [87].

Our results highlight how choices in numerical implementation affect parameter estimates and suggest several directions for future work that may improve our approach. For example, the optimal value of our parameter controlling the time scale of cell migration ($\alpha_{\mathrm{MM}}$) appears to be independent of the initial condition and the mesh resolution that we used to construct PDE solutions, provided the latter is sufficiently refined. One drawback of our current approach, however, is that we may need to estimate $\alpha_{\mathrm{MM}}$ and *γ* for each new choice of discrete rules governing migration and cell birth, respectively, because these rules perturb the short-range interactions between relatively small numbers of cells. This naturally leads to the question of whether an analytic expression can be derived for these parameters. Several coarse-graining techniques that take into account higher-order correlations between cells in on- or off-lattice models may produce scaling factors similar to those introduced in this paper, but these may only apply to certain classes of ABMs [88]. Alternatively, it may be possible to estimate scaling parameters of continuous models by investigating the convergence of EA ABM results to features of their solutions such as the speed of solution propagation, as in [85,86] for on-lattice models; by accounting for the dynamics of the two-particle distribution, as in [73,74]; or by exploring scaling relationships as in [89]. Adapting these approaches to our setting is an interesting avenue for future work.

Additionally, our continuous models more accurately represent ABM results within the bulk of the solution support because the *L*^2^ norm more strongly penalizes discrepancies there. In the future, other norms, such as the *L*^∞^ error, could be used to match the solution supports given by our discrete and continuous models. Replacing our birth-rate scaling parameter *γ* with a density-dependent function—through either rigorous derivation or an equation-learning approach [90]—is another exciting future direction. In particular, because cell proliferation in the ABM involves selecting positions uniformly at random from the domain each day, the chance that we select a location **z** that permits birth appears to depend in a nonlinear way on the solution support. More generally, our computational study does not provide theoretical explanations for our parameter values, and we plan to build on the intuition that we established here to develop these arguments in the future.

To simplify our initial study, we considered the dynamics of one cell population (i.e. melanophores in the main text and xanthophores in electronic supplementary material, S6), but pattern formation in zebrafish skin involves multiple cell types and longer-range interactions, as we highlight in electronic supplementary material, figure S1. Future work may extend our pipeline to construct more realistic continuous models with multiple cell types and interaction neighbourhoods. Related to this, the initial conditions that we designed allowed us to make one-to-one comparisons between discrete- and continuous-model densities, but this may not always be possible. More realistic zebrafish models (i.e. [14,37,39,40]) produce patterns that are more complicated than our box and stripe motifs. This means that ensemble-averaging stochastic ABM realizations may not retain information about the length scales inherent in patterns. For such cases, fitting parameters based on summary statistics (e.g. pair-correlation functions [91], pattern-simplicity scores [92] or persistent-homology approaches [93]) may be more useful, and we plan to address this in future work. These and other directions move us toward constructing interpretable, analytically tractable continuous models of self-organization, increasing our understanding of biological pattern formation more broadly.

## Data Availability

Data and relevant code for this research work are stored in GitHub: https://github.com/wdmartinson/Self-Organization-One-Species/tree/first_version and have been archived within the Zenodo repository: https://doi.org/10.5281/zenodo.11160930 [94]. Electronic supplementary material, figures and videos are available on Figshare at https://figshare.com/projects/Linking_discrete_and_continuous_models_of_cell_birth_and_migration_in_one_population/171234. Raw data used to generate figures in the main text and electronic supplementary material have been archived in the Dryad repository https://doi.org/10.5061/dryad.s4mw6m9cb [95]. Supplementary material is available online [96].
